# A High-Carbohydrate Diet Prolongs Dysbiosis and Clostridioides difficile Carriage and Increases Delayed Mortality in a Hamster Model of Infection

**DOI:** 10.1128/spectrum.01804-21

**Published:** 2022-06-16

**Authors:** Shrikant S. Bhute, Chrisabelle C. Mefferd, Jacqueline R. Phan, Muneeba Ahmed, Amelia E. Fox-King, Stephanie Alarcia, Jacob V. Villarama, Ernesto Abel-Santos, Brian P. Hedlund

**Affiliations:** a School of Life Sciences, University of Nevada, Las Vegas, Las Vegas, Nevada, USA; b School of Public Health, University of Nevada, Las Vegas, Las Vegas, Nevada, USA; c Department of Chemistry and Biochemistry, University of Nevada, Las Vegas, Las Vegas, Nevada, USA; d College of Osteopathic Medicine, Touro University, Nevada, Henderson, Nevada, USA; e Kirk Kerkorian School of Medicine, University of Nevada, Las Vegas, Las Vegas, Nevada, USA; f Nevada Institute of Personalized Medicine, University of Nevada, Las Vegas, Las Vegas, Nevada, USA; University of California, Davis

**Keywords:** Clostridioides difficile, carbohydrates, diet, microbiome

## Abstract

Studies using mouse models of Clostridioides difficile infection (CDI) have demonstrated a variety of relationships between dietary macronutrients on antibiotic-associated CDI; however, few of these effects have been examined in more susceptible hamster models of CDI. In this study, we investigated the effect of a high-carbohydrate diet previously shown to protect mice from CDI on the progression and resolution of CDI in a hamster disease model, with 10 animals per group. Hamsters fed the high-carbohydrate diet developed distinct diet-specific microbiomes during antibiotic treatment and CDI, with lower diversity, persistent C. difficile carriage, and delayed microbiome restoration. In contrast to CDI protection in mice, most hamsters fed a high-carbohydrate diet developed fulminant CDI including several cases of late-onset CDI, that were not observed in hamsters fed a standard lab diet. We speculate that prolonged high-carbohydrate diet-specific dysbiosis in these animals allowed C. difficile to persist in the gut of the animals where they could proliferate postvancomycin treatment, leading to delayed CDI onset. This study, along with similar studies in mouse models of CDI, suggests some high-carbohydrate diets may promote antibiotic-associated dysbiosis and long-term C. difficile carriage, which may later convert to symptomatic CDI.

**IMPORTANCE** The effects of diet on CDI are not completely known. Here, we used a high-carbohydrate diet previously shown to protect mice against CDI to assess its effect on a hamster model of CDI and paradoxically found that it promoted dysbiosis, C. difficile carriage, and higher mortality. A common thread in both mouse and hamster experimental models was that the high-carbohydrate diet promoted dysbiosis and long-term carriage of C. difficile, which may have converted to fulminant CDI only in the highly susceptible hamster model system. If diets high in carbohydrates also promote dysbiosis and C. difficile carriage in humans, then these diets might paradoxically increase chances of CDI relapse despite their protective effects against primary CDI.

## INTRODUCTION

Clostridioides difficile infection (CDI) is caused by toxigenic strains of the anaerobic, Gram-positive, spore-forming enteropathogen *Clostridioides* (formerly *Clostridium*) *difficile* (1). CDI is the most commonly identifiable cause of antibiotic-associated diarrhea. The incidence of CDI is complicated by the appearance of highly resistant and hypervirulent BI/NAP1/027 strains that have an attributable mortality of an astonishing 22% (2). In the United States, hospital- and community-acquired CDI accounted for 223,900 cases with 12,800 deaths in 2017 (3), leading to a significant financial burden ($4.8 billion) on the U.S. health care system (3–5).

The outcome of CDI varies widely from asymptomatic colonization to mild and self-limiting diarrhea, to severe diarrhea and life-threatening pseudomembranous colitis (1, 6). The diverse and unpredictable CDI outcomes can be linked to both C. difficile-specific and host-related factors. C. difficile pathogenicity loci vary considerably from strain to strain (7, 8), leading to the variable presence of pathogenesis factors. For example, the presence of the two large clostridial toxins (TcdA and TcdB) is strain-dependent and can be attributed to different CDI outcomes (9). C. difficile is also catabolically versatile and uses both diet- and host-derived amino acids and sugars during the course of infection (10). In addition, host-derived primary bile acids act as spore cogerminants that can amplify C. difficile growth in the gut (11). Yet, under normal circumstances, the normal gut flora provides colonization resistance to acute C. difficile infections (12), likely due to niche exclusion and the production of inhibitory volatile fatty acids and secondary bile acids (13). When the microbiota is disturbed (e.g., antibiotic treatment) C. difficile spores can germinate and establish infection.

Although diet is known to modulate gut microbial ecology (14, 15), studies on the effects of dietary macronutrients on CDI outcome have been largely restricted to small animal models, mostly mice (16–18). Recent studies conducted using mouse models of antibiotic-associated CDI have shown that high-protein diets lead to fulminant CDI and high mortality rates (16–18). These studies indicate some degree of consensus on the effect of protein-rich diets leading to severe CDI, but the effects of dietary carbohydrates appear to be more complex. For example, diets high in microbiota-accessible carbohydrates (MACs) that are fermented to short-chain fatty acids in the intestinal tract have been reported to be protective against CDI (17). Conversely, other studies have implicated simple carbohydrates, specifically trehalose, in the proliferation of hypervirulent C. difficile strains (19) and dietary glucose and fructose in enhancement of both spore production and host colonization capacity (20). More recently, it was shown that both sorbitol (21) and lactotrehalose (22) can reduce toxin production *in vivo*.

In contrast, our previous study showed that a diet rich in carbohydrates decreased CDI severity and eliminated mortality in mice, despite the fact that the carbohydrates provided in the diet were readily digestible sucrose and starch (18). Interestingly, our study also indicated that the high-carbohydrate diet led to an asymptomatic persistent carrier state in which C. difficile was present in the murine gut for at least a month after primary CDI was resolved, with constant shedding into feces.

Our previous study and other similar studies have been carried out using a recently developed mouse model of CDI (23–25), which is more resistant to the development of CDI over the more traditional, highly sensitive hamster model (26). For over 5 decades, Syrian golden hamster models of CDI have been extensively used to assess the growth dynamics and pathophysiology of C. difficile, the susceptibility and severity of CDI following different antibiotic regimens, and the efficacy of various treatment options (26–29). Hamsters are exquisitely sensitive to CDI and develop fulminant colitis within 48-h postchallenge. These extreme symptoms are rarely seen in primary human CDI. However, hamster CDI severity can be modulated by vancomycin treatment, which is also used clinically to treat CDI. Indeed, suboptimal vancomycin treatment of infected hamsters results in delayed sign onset, similar to human CDI relapse, and also prevents non-CDI clindamycin-associated colitis (30). Previously, dietary supplementation of fructooligosaccharides (FOS) and soy fiber have been shown to delay CDI onset, attenuate CDI development, and increase survival time in hamsters (31, 32). Additionally, increased CDI susceptibility has been observed in hamsters fed an atherogenic diet (33). Despite these efforts, the effects of dietary macronutrients on CDI in hamster models of disease are not completely understood. Considering the lack of clinically translatable human studies indicating the precise effect of dietary macronutrients on CDI outcome, and the lack of consensus on the effects of carbohydrates on CDI in murine models (17–20), a better understanding of the effects of dietary carbohydrates on hamster models of CDI is likely to provide useful insights into C. difficile pathogenesis.

Here, we modulated the hamster model of CDI with subclinical vancomycin doses. This model was then used to assess the effect of a high-carbohydrate diet previously shown to be protective in mice (18). The murine CDI model shows graded symptom progression that better mimics human primary CDI. In contrast, vancomycin treatment of infected hamsters results in delayed severe sign onset similar to human CDI relapse. In hamsters, this high-carbohydrate diet promoted antibiotic- and CDI-associated dysbiosis and C. difficile carriage, eventually leading to higher mortality after completion of vancomycin pretreatment. Thus, although some high-carbohydrate diets can prevent severe CDI in some settings, they may also promote C. difficile carriage, which could change from an asymptomatic carrier state to severe CDI relapse depending on environmental cues.

## RESULTS

### The high-carbohydrate diet exacerbated antibiotic- and CDI-associated microbiome diversity loss.

**FIG 1:**
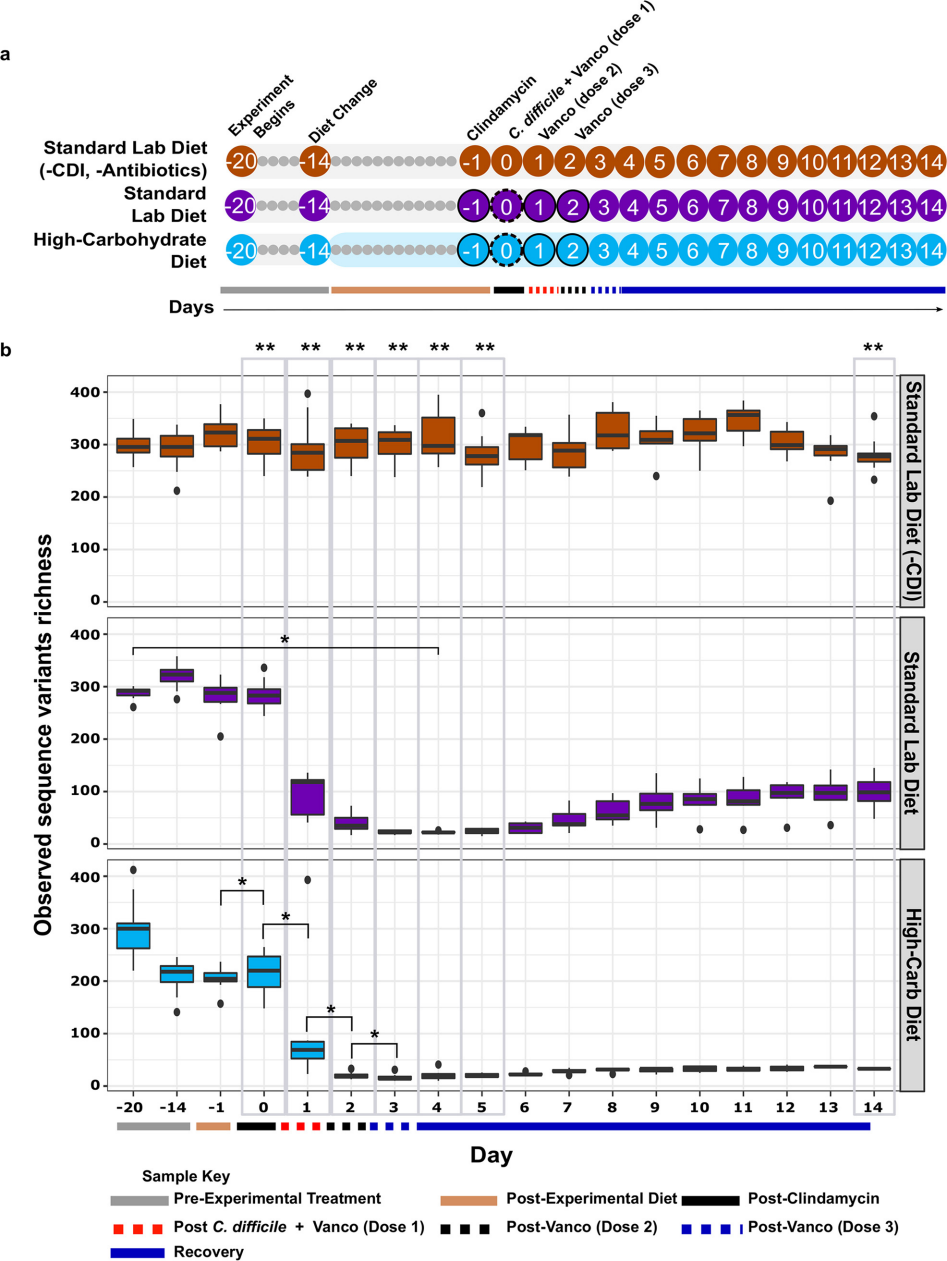
Experimental timeline and effect on fecal microbiome richness. (a) Animals were fed a standard lab diet and from day −20 to −14, at which time the high-carbohydrate diet was introduced to the high-carbohydrate diet group and was maintained for the remainder of the experiment. Clindamycin was administered on day −1 to induce dysbiosis, and hamsters were challenged with C. difficile R2027 spores on day 0. A vancomycin regimen (outline circle) to mitigate CDI was carried out on days 0 to 2. Circles with numbers indicate days fecal samples were collected. Stool collection took place before the manipulation of hamsters or experimental treatment. Ten animals per group in individual cages. (b) Observed sequence variants is shown for uninfected hamsters fed a standard lab diet (orange, *n* = 10), infected hamsters fed a standard lab diet (purple, *n* = 9), and infected hamsters feed a high-carbohydrate diet (blue, *n* = 10). Administration of experimental diets (solid tan line, *x* axis), time points after antibiotics (dashed lines, *x* axis), and C. difficile challenge (black line, *x* axis) are indicated. Black dots above and below boxplots represent outliers. (*) Indicates significant (*P* < 0.05) loss of diversity in within-group pairwise comparisons shown by the brackets (two-way mixed ANOVA with Bonferroni correction). Gray boxes highlight comparisons between groups after a change in diet on day −1, antibiotic treatments on days 0 to 3, postinfection on days 4 and 5, and recovery on day 14. (**) Significant (*P* < 0.05) difference between groups on a given day (repeated measures mixed ANOVA followed by Tukey’s HSD test). The same sample key from Fig. 1 is shown on most of the following figures for clarity.

Over 13.9 million high-quality 16S rRNA gene fragment reads, representing 3,533 unique amplicon sequence variants (ASVs), were obtained from 447 fecal samples collected along the experimental timeline (Fig. 1a). To understand changes in the fecal microbiome due to experimental diets within the context of the CDI time course, alpha diversity was analyzed using observed ASVs (Fig. 1b), Simpson’s evenness (Fig. S2), and Shannon diversity (Fig. S3). Untreated, uninfected control hamsters without antibiotics and fed the standard lab diet (standard lab diet [-CDI] group) showed no significant change in alpha-diversity metrics over time (*P* > 0.05). In contrast, there were significant changes for all three diversity metrics over time (*P* < 0.05) for both experimental groups. In the standard lab diet group, this constituted a >90% reduction of observed ASVs during antibiotic treatments and CDI compared with preantibiotic levels (Fig. 1b). For these hamsters, ASVs richness increased in surviving animals by the end of the experiment, but was still well below a preantibiotic, preinfection state at the end of the experiment (Fig. 1b, day −1 mean 282.8 ± 34.51 S.D. versus day 14 mean 98.5 ± 34.0 S.D.). This effect was exaggerated for animals in the high-carbohydrate diet group, where mean community richness was depleted by >98% by day 2 (Fig. 1b, day −1 mean 205.2 ± 21.7 S.D. versus day 2 mean 19.9.0 ± 5.9 S.D., *P* < 0.05) and did not rebound for the duration of the experiment. There were also significant daily decreases in diversity in the high-carbohydrate diet group microbiomes after clindamycin administration (day −1 versus day 0), after the first dose of vancomycin and C. difficile challenge (day 0 versus day 1), after the second dose of vancomycin (day 1 versus day 2), and after the final dose of vancomycin (day 2 versus day 3) (*P* < 0.05, two-way repeated-measures ANOVA). There were no significant differences in observed ASVs between the standard lab diet and high-carbohydrate diet groups from days 1 to 5 (post-CDI) (*P*-value > 0.05, Tukey’s HSD), but ASV richness was significantly lower at the end of the experiment in the high-carbohydrate fecal microbiome (*P*-value < 0.05, Tukey’s HSD) (Fig. 1b).

### Antibiotics and CDI disrupted microbial community composition, which was prolonged in animals fed the high-carbohydrate diet.

**FIG 2:**
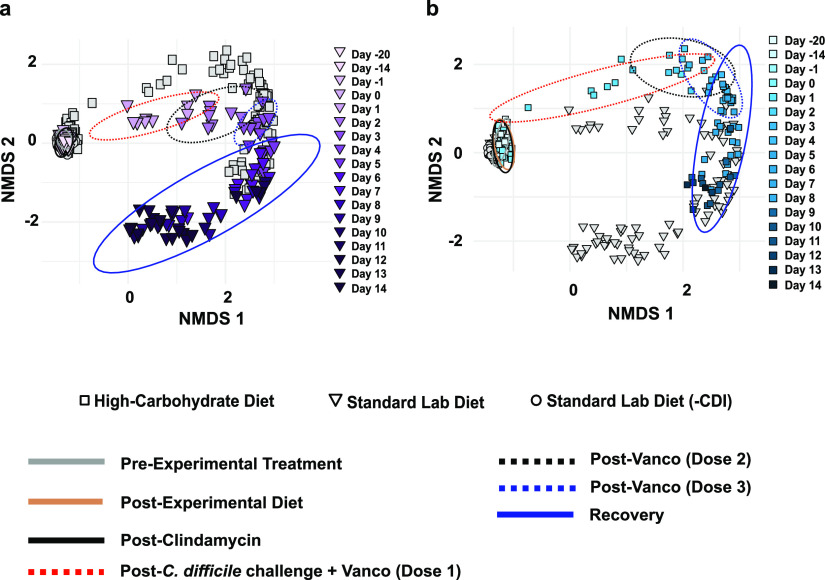
NMDS analysis based on Bray-Curtis dissimilarity. Each panel is a visualization of the same data and highlights the analysis for infected hamsters fed (a) the standard lab diet (purple, triangles) and (b) the high-carbohydrate diet (blue, squares) beginning on day −14, continuing for the duration of the experiment. Colors are shaded to show time progression through the experiment. Ellipses represent standard errors of the mean (95% confidence) for samples associated with the standard lab diet (pre-experimental treatment, gray solid line, days −20, −14, −1), the diet-specific microbiomes (postexperimental treatment, tan solid line, day −1), the effect of clindamycin (post-Clindamycin, black solid line, day 0), the first dose of vancomycin and CDI challenge (post-C. difficile challenge + Vanco [dose 1], red dashed line, day 1), the second dose of vancomycin (post-Vanco [dose 2], black dashed line, day 2), the third dose of vancomycin (post-Vanco [dose 3], blue dashed line, day 3), and the recovery phase (solid blue line, days 4 to 14).

To further assess microbial community alterations over time, Bray-Curtis dissimilarity was visualized by nonmetric multidimensional scaling (NMDS) (Fig. 2; Fig. S3). Preantibiotic diet-specific communities did not emerge, as indicated by overlapping “pre-experimental” (gray solid line) and “postexperimental diet” (tan solid line) confidence ellipses (ANOSIM *R* = 0.15, *P*-value = 0.18) (Fig. S4). In the experimental groups (Fig. 2), the microbial communities were disrupted by antibiotic treatments and CDI, as indicated by progressive shifts in ellipses associated with the 3-day vancomycin regimen and C. difficile spore challenge (red, black, and blue dashed ellipses). Moreover, the vancomycin treatment- and CDI-associated ellipses were distinct between hamsters based on diet, demonstrating either a progression of changes to the microbiome or a synergistic interaction between diet and antibiotic treatment/CDI that changed the gut microbiome composition through the full course of the experiment: post-C. difficile challenge and vancomycin dose 1 (red ellipses, ANOSIM: *R* = 0.5, *P* = 0.001); postvancomycin dose 2 (black ellipses, ANOSIM: *R* = 0.64, *P* = 0.001); postvancomycin dose 3 (blue ellipses, ANOSIM: *R* = 0.89, *P* = 0.001); and recovery phase (blue ellipses, ANOSIM: *R* = 0.57, *P* = 0.001). Full microbiome recovery was not seen in surviving animals in either experimental group, though the microbiomes in the standard lab diet group approached the pre-experimental condition, whereas those in the high-carbohydrate diet group did not change beyond the acute phase of CDI (blue ellipses). The microbiomes in the negative control group (standard lab diet [-CDI]) did not vary throughout the experiment (Fig. S3).

**FIG 3:**
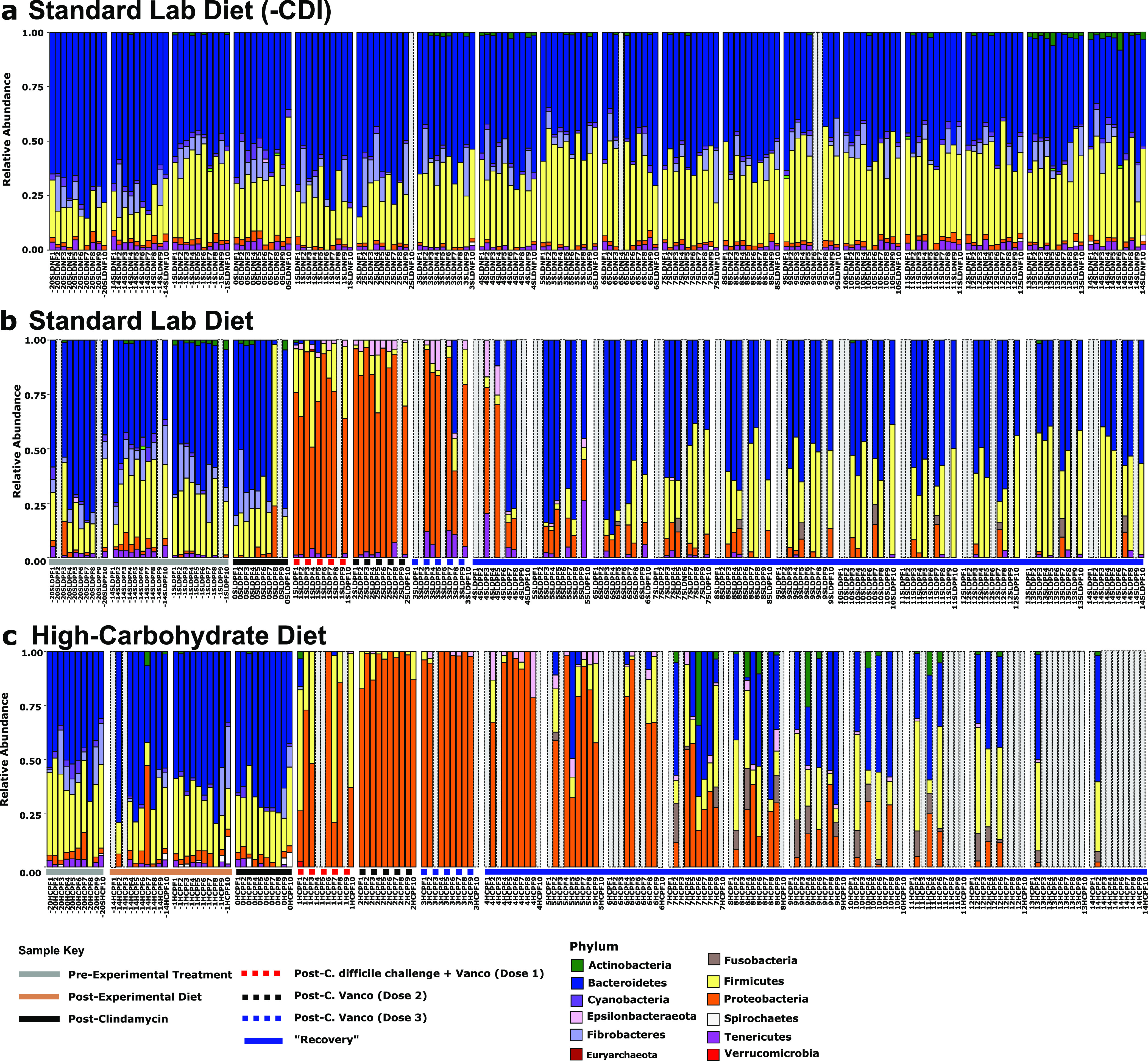
Phylum relative abundance bar plots. Bar plots showing the relative abundance of fecal microbiota at the phylum level in each hamster throughout the experiment. (a) standard lab diet (-CDI); (b) standard lab diet; (c) high-carbohydrate diet. Animals for which data are missing due to mortality, missing data due to rarefaction, or statistical outlier are indicated by a gray bar.

These changes in beta diversity were consistent with changes in phylum-level relative abundance, which showed dramatic shifts in the microbiomes for both experimental groups beginning on day 1 (Fig. 3). These changes are characterized by large expansions of *Proteobacteria* in both experimental groups, which coincided with antibiotic treatment and acute CDI. The high abundance of *Proteobacteria* largely subsided by day 5 in the standard lab diet group microbiomes (Fig. 3b) but persisted in the high-carbohydrate diet group microbiomes (Fig. 3c). There were smaller expansions of *Epsilonproteobacterota* (specifically *Campylobacteraceae*) coincident with antibiotic treatment and acute CDI in both experimental groups, which similarly persisted longer in the high-carbohydrate diet group microbiomes (Fig. 3c). Only the standard lab diet group microbiomes showed a transient increase in *Tenericutes* during CDI, which quickly decreased to pre-experimental conditions. In contrast, *Tenericutes* became depleted during antibiotic treatment and acute CDI in the high-carbohydrate diet group microbiomes and never recovered. *Fibrobacteretes* became depleted during antibiotic treatment and acute CDI in both experimental groups and also never recovered. Some animals in both experimental groups showed increases in *Fusobacteria* during recovery from acute CDI, and some animals in the high-carbohydrate diet group showed increases in *Actinobacteria*. The microbiomes in the negative control group (standard lab diet [-CDI]) did not vary throughout the experiment (Fig. 3a).

### Diet-specific microbiome changes were associated with antibiotic treatments and CDI and recovery.

**FIG 4:**
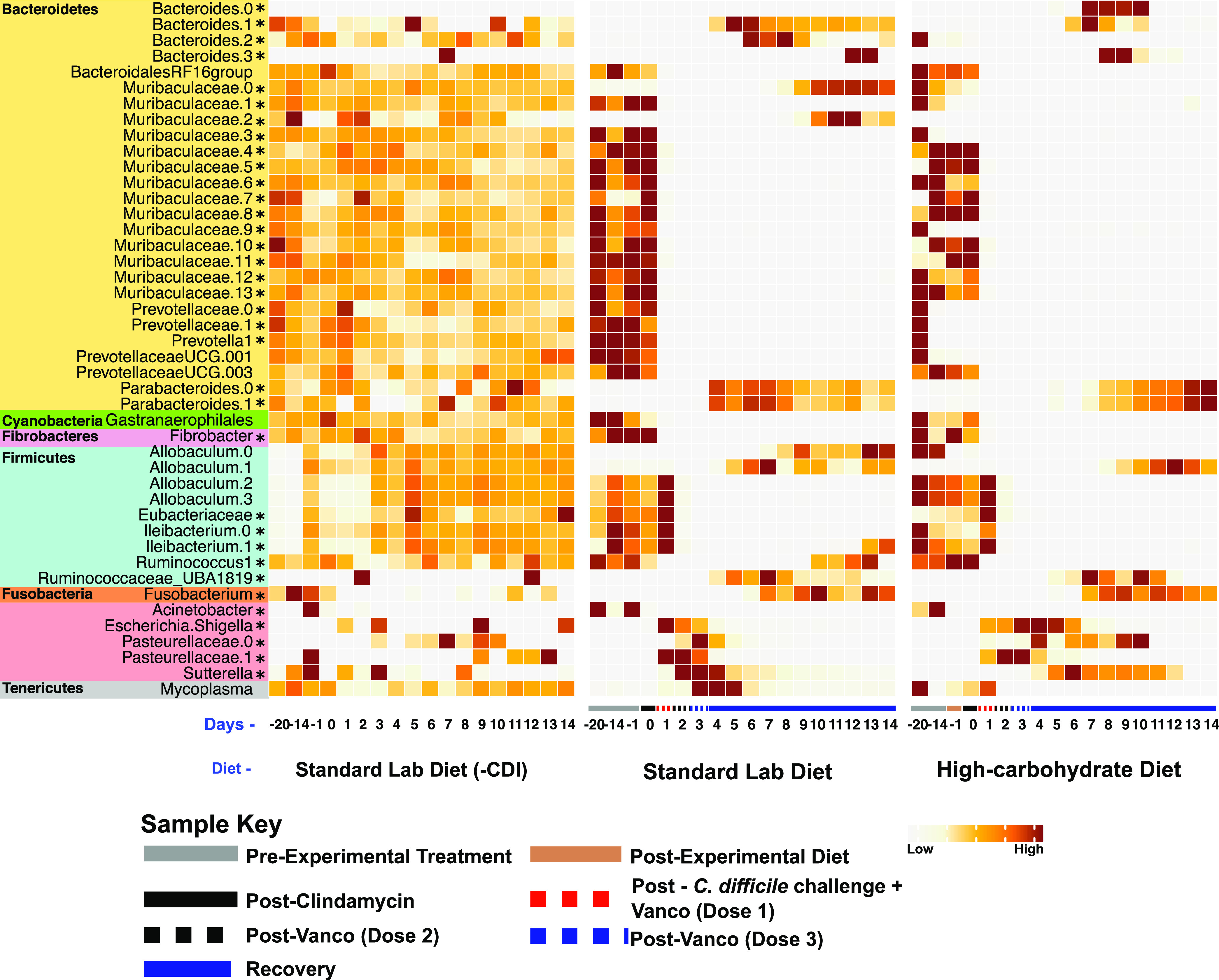
Heatmap of 44 ASVs identified using SIMPER analysis that contributed to 30% of observed dissimilarity. Results display top ASVs responsible for dissimilarity between experimental groups. The heat map indicates the mean relative abundances of 44 ASVs that contributed cumulatively to 30% of community dissimilarities at each time point among the hamsters fed the standard laboratory diet and high-carbohydrate diet. Each square represents the mean relative abundance of the given ASV on a particular day for a particular diet. Higher intensity of brown coloring correlates with higher relative abundance, compared with the relative abundance of the same ASV throughout that experimental treatment. Asterisks (*) indicate taxa discussed in the manuscript text.

SIMPER analysis was performed to identify ASVs that contributed most to the observed changes in beta diversity (Fig. 4). This analysis identified 44 ASVs that cumulatively accounted for 30% of microbial community dissimilarity between all pairwise comparisons of the three animal groups throughout the experiment. Almost one-third of these ASVs (13 ASVs) belonged to the family *Muribaculaceae*. Nearly all *Muribaculaceae* ASVs decreased in abundance after administration of the first dose of vancomycin and C. difficile spores, with recovery of one of the *Muribaculaceae* ASVs (*Muribaculaceae.*0) and emergence of a new *Muribaculaceae* ASV (*Muribaculaceae.*2) only in hamsters in the standard lab diet group late in the experiment.

Several other taxa also decreased following antibiotics and CDI in both experimental groups, with some showing diet-specific responses. For example, one *Ileibacterium* ASV (*Ileibacterium.1*) and *Ruminococcus* recovered only in the standard lab diet group. Several *Prevotellaceae* ASVs were similarly depleted following antibiotic treatment and CDI in the standard lab diet group, but they were already depleted following the change in diet in the high-carbohydrate group before antibiotic treatment. None of these *Prevotellaceae* ASVs recovered during the experiment. Similarly, an unassigned member of the *Gastranerophilales* within the *Cyanobacteria*, a member of the genus *Fibrobacter* within the *Fibrobacteres*, and several ASVs assigned to the *Firmicutes* (i.e., *Eubacteriaceae* [one ASV], one ASV belonging to *Ileibacterium* [two ASVs], and an unassigned *Eubacteriaceae* ASV [one ASV]) were depleted due to antibiotics and CDI and never recovered.

Other groups increased in abundance in response to dysbiosis. There was a transient increase after antibiotic administration in members of the phylum *Proteobacteria*, which included ASVs assigned to Acinetobacter (one ASV), Escherichia/*Shigella* (one ASV), *Pasteurellaceae* (two ASVs), and *Sutterella* (one ASV). Outside the *Proteobacteria*, some other taxa showed similar patterns, including *Parabacteroides* (two ASVs), *Fusobacterium* (one ASV), and *Mycoplasma* (one ASV). Four members of the genus *Bacteroides* also expanded during dysbiosis but showed diet-specific patterns. *Bacteroides.0* expanded only in the high-carbohydrate diet group, whereas *Bacteroides.2* expanded only in the standard lab diet group. The two other *Bacteroides* ASVs expanded in both experimental groups.

### The high-carbohydrate diet prolonged C. difficile carriage.

**FIG 5:**
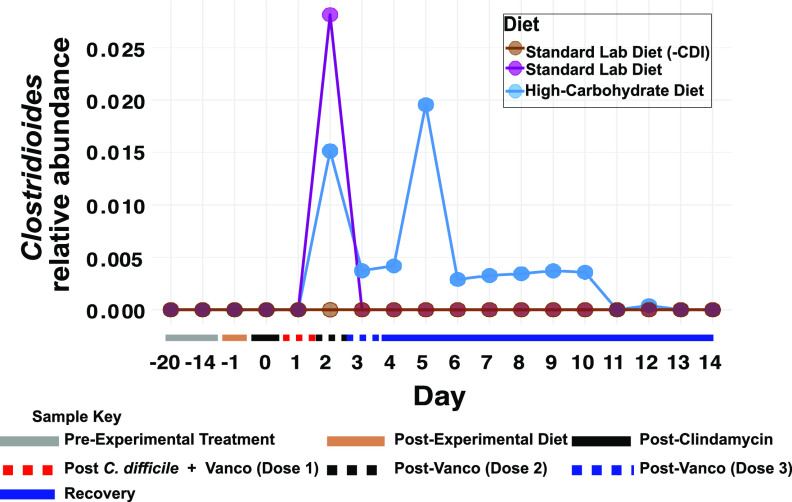
C. difficile relative quantification. The 16S rRNA amplicon-based relative abundance of C. difficile across the experimental timeline. Each dot represents the mean relative abundance of C. difficile on a particular day for a given diet.

Relative quantification of C. difficile in fecal microbiomes demonstrated prolonged carriage in the high-carbohydrate diet group. The relative abundance of C. difficile among ASVs was similar in the two experimental groups on day 2 (2 days postinfection) (Fig. 5). However, C. difficile dropped below the detection limit for all surviving hamsters in the standard lab diet microbiomes by day 3 and remained undetectable for the remainder of the experiment. In contrast, C. difficile remained detectable in surviving hamsters in the high-carbohydrate diet group from day 2 to day 10.

### The high-carbohydrate diet increased secondary CDI and mortality in hamsters.

**FIG 6:**
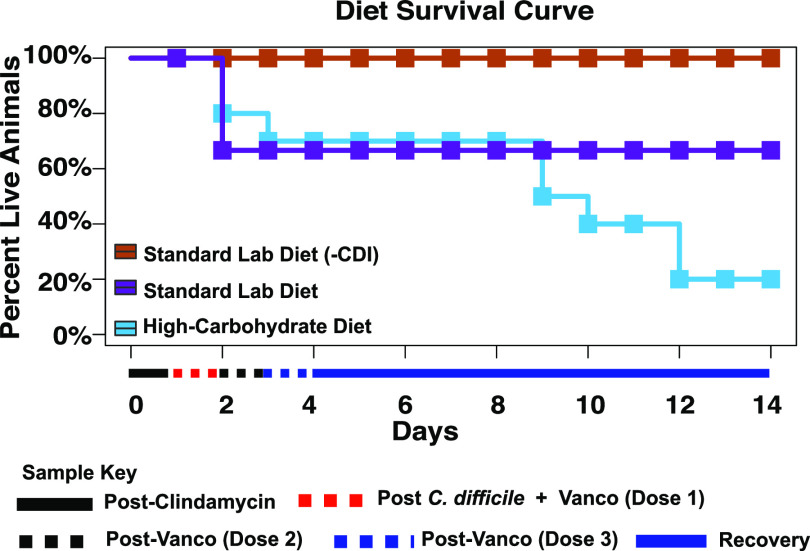
Effect of diet on hamster survival. Kaplan-Meier survival curves for uninfected hamsters fed the standard lab diet and without antibiotics (orange, *n* = 10), and infected hamsters fed the standard lab diet (purple, *n* = 9) and high-carbohydrate diet (blue, *n* = 10). There were no significant reductions in survival (*P* > 0.05, log-rank test) between the two experimental groups for the full experiment or for the acute phase of CDI (days 0 to 4); however, there was a significantly higher mortality in the high-carbohydrate group later in the experiment due to delayed-onset CDI (days 5 to 15; *P* = 0.01, log-rank test).

Hamster mortality was documented to assess the effect of the high-carbohydrate diet on hamster survival after treatment with subclinical concentrations of vancomycin and challenge with C. difficile spores on day 0 until the end of the experiment on day 14 (Fig. 6). Three independent statistical tests were run to assess mortality. Although differences in the full experimental course or the acute phase of CDI (days 1 to 4) were not statistically significant (*P* > 0.05, log-rank test), there was a significant difference between mortality in the later phase of CDI (days 5 to 14; *P* = 0.051, log-rank test), documenting a higher incidence of mortality due to delayed-onset CDI in animals fed the high-carbohydrate diet. Three of the nine hamsters fed the standard lab diet and infected with C. difficile developed severe CDI and were euthanized after 48 h of infection (33% mortality) during the acute phase of CDI, while the remaining six hamsters survived for the entirety of the experiment. In comparison, two of the 10 hamsters fed the high-carbohydrate diet and infected with C. difficile developed severe CDI during the acute phase and were euthanized after 48 h of infection; one more hamster died within the next 24 h, after which no mortality was observed until day 8. Five additional hamsters fed the high-carbohydrate diet died on day 9 (two hamsters), day 10 (one hamster), and day 12 (two hamsters), leading to a total of 80% mortality of the hamsters fed the high-carbohydrate diet. All control hamsters fed the standard lab diet but not infected (standard lab diet [-CDI]) survived for the duration of the experiment.

## DISCUSSION

Syrian golden hamsters have been instrumental as experimental models to understand various aspects of C. difficile biology and pathogenesis; however, the effects of diet on CDI in hamster models of disease have not been fully explored. Here, a standard lab diet and a high-carbohydrate diet containing simple sugars were directly compared in an antibiotic-associated CDI model in hamsters to explore their effects on CDI, hamster mortality, and microbial community dynamics.

### Effect of antibiotics, CDI, and diet on fecal microbiomes in hamsters.

Conventionally, reduced fecal microbial diversity is considered a hallmark of CDI susceptibility and the loss of colonization resistance (34, 35). However, more recent reports indicate CDI susceptibility is not specifically related to microbial diversity and suggest that diet plays a more important role in CDI outcome (17, 18). For example, the microbial-accessible carbohydrate inulin was shown to increase short-chain fatty acid production, reduce C. difficile burden, and promote CDI resolution in a mouse model of disease, with no significant effect on microbiome diversity (17). More strikingly, in our previous study using mice, we showed that a high-carbohydrate diet rich in sucrose and digestible starch dramatically reduced microbiome richness and diversity during antibiotic treatment and CDI compared with mice on the standard lab diet; however, that diet prevented CDI signs such as anogenital discoloration, wet tail, hunchback posture, lethargy, diarrhea, and loss of body weight in almost all animals, and those that showed mild CDI signs quickly recovered. Here, we observed a similar effect of the high-carbohydrate diet on microbial diversity in hamsters (Fig. 2**;** Fig. 3). However, in this case lower microbial diversity correlated with poor long-term prognosis (Fig. 6).

Despite overall similar effects of this high-carbohydrate diet on the microbiomes in mouse and hamster experimental models, the dynamics of these microbial communities were distinct. For example, mice developed distinct microbiomes in response to the high-carbohydrate diet before antibiotic treatment and C. difficile challenge. That change was related to depletion of several members of the *Lachnospiraceae* and *Ruminococcaceae*. Although pre-CDI, diet-specific microbiomes were not evident in hamsters (Fig. 3), a depletion of some *Prevotellaceae* was observed following the change in diet (Fig. 4). In both models and both diets, antibiotic treatment and CDI was accompanied by a proliferation of *Proteobacteria*. Many gut *Proteobacteria*, particularly *Enterobacteriaceae*, overgrow in response to inflammation, due to respiration of reactive nitrogen species-derived nitrate and nitrite (by denitrification and dissimilatory nitrite reduction to ammonium), which promotes a proinflammatory positive feedback loop (36). In this study, a more diverse group of respiratory *Proteobacteria* expanded during dysbiosis and CDI (compared with results in mice), which may be related to the more sensitive nature of hamsters to CDI and/or the effects of vancomycin, which was not used in the mouse CDI model. Blooms of *Proteobacteria* in the dysbiotic gut have been documented in many microbiome studies performed in humans and mice with CDI (37, 38).

A very striking response to antibiotics and CDI in hamsters was the depletion of many members of the *Muribaculaceae*, making up almost 30% of ASVs that significantly changed through the course of the experiment (Fig. 4). Although very few members of the *Muribaculaceae* have been cultivated in the laboratory, both metagenomics and cultivation studies show that they have an abundance of glycoside hydrolases and ferment MACs to SCFAs (39–43). The fact that many members of the *Muribaculaceae* were greatly reduced during CDI in hamsters suggests a loss of SCFA production, which has been associated with decreased gut health due to changes in gut epithelia morphology and permeability, and poor CDI outcomes (17, 44–46). However, the expansion of some other *Muribaculaceae* during and after CDI in the standard lab diet group complicates this interpretation and begs for more detailed study of individual species and strains in the *Muribaculaceae*. There was also a decrease in relative abundance of members of the phyla *Cyanobacteria*, *Fibrobacteres*, *Firmicutes*, and *Tenericutes* after administration of antibiotics regardless of diet (Fig. 3b, c; Fig. 4). This loss in specific taxa due to antibiotic treatment is consistent with previous research in mice (35, 47).

The microbiome in standard lab diet was somewhat restored by the end of the experiment (Fig. 2a, 3b), as evidenced by recovery of some ASVs, including some members of the genera *Bacteroides*, *Ruminococcus*, *Ileibacterium*, and *Muribaculaceae* (Fig. 4). The appearance of these ASVs during the recovery phase was also reflected NMDS plots based on Bray-Curtis dissimilarity showing the day 14 microbiomes approaching the “pre-experimental treatment” time points for standard lab diet microbiomes (Fig. 2a). This provides evidence of partial recovery of the gut microbial community after antibiotic-induced CDI, which is consistent with studies that examine the effect diet on CDI outcomes in mice.

### Simple sugars promote C. difficile carriage in animal models and may later become symptomatic.

Despite similar CDI courses during the acute phase (within 72 h of infection), the high-carbohydrate diet led to a poor prognosis, particularly due to delayed-onset CDI later in the experiment (Fig. 6). To our knowledge, dietary carbohydrates have not previously been shown to exacerbate CDI in any animal models. We propose that this unusual result was a product of prolonged dysbiosis and C. difficile carriage due to effects of antibiotics, the high-carbohydrate diet, and the extreme sensitivity of hamsters to CDI.

In this study, C. difficile was detectable in the high-carbohydrate diet group microbiomes by day 2 and remained so for most of the experiment (Fig. 5). Strikingly, C. difficile was not detected in the standard lab diet amplicon data set after day 2, despite the deep sequencing effort. Microbiome richness was severely depleted (>98% reduction in ASVs [Fig. 1b]) by day 2 in these animals and did not increase significantly for the duration of the experiment. Hamsters in the high-carbohydrate diet suffered from a second period of mortality triggered days after a second C. difficile bloom was detected. Similarly, Bray-Curtis analyses showed that high-carbohydrate diet microbiomes remained dysbiotic throughout the experiment (Fig. 2b) and many ASVs did not recover (Fig. 4). Very similar microbiome changes were seen previously using these exact same diets in mice (18), where C. difficile persisted in animals fed the high-carbohydrate diet but appeared to be cleared from mice on the standard lab diet by the end of the experiment, 30 days after C. difficile challenge. Similarly, final microbiome richness was >2X higher for mice fed the standard lab diet compared to the high-carbohydrate diet and the high-carbohydrate diet microbiome was never fully restored (Fig. 2).

Despite similar microbiome responses, the outcomes of CDI in hamsters and mice on these two diets were opposite. We propose that these different outcomes relate to the tolerance of these two animals to C. difficile. Although hamster models of CDI mirror the classic features of human CDI including the shifts in gut microbial communities, diarrhea and histological anomalies, and more advanced changes such as development of pseudomembranes, it is very different from murine models of CDI. Hamsters are highly susceptible to CDI; they show severe and sudden onset of CDI symptoms with as little as a few dozen spores (48, 49) and require suboptimal doses of vancomycin to prevent C. difficile overgrowth and death (49, 50). In contrast, mice are resistant to CDI development and require a greater spore load (~10^8^ spores), an aggressive antibiotic regimen, and are able to recover from infection within a few days (24, 51–54). Mice models are also slow to develop CDI and display a wide range of CDI signs (24, 55). We therefore speculate that inherent differences between host susceptibility to CDI led to the observed differences in the CDI outcome (18). In the case of the hamster experiments described here, we propose that vancomycin administered to animals on days 0 to 3 limited C. difficile proliferation until the vancomycin was cleared from the system, after which C. difficile proliferated and led to severe, acute CDI, and death beginning on day 8.

### Conclusions.

This study, along with our recent study in a mouse CDI model, documented persistent dysbiosis in animals fed a high-carbohydrate diet following antibiotic treatment and CDI. The dysbiotic, high-carbohydrate diet microbiome was conducive to prolonged C. difficile carriage in both experimental systems. While mice on the high-carbohydrate diet were protected from CDI and persisted in an asymptomatic carrier state, C. difficile proliferated in hamsters following termination of vancomycin treatment, leading to higher mortality in the high-carbohydrate diet group. The divergent long-term prognoses of the two experimental models in response to the high-carbohydrate diet may translate to different diet-specific responses in humans. Additionally, if high-carbohydrate diets promote asymptomatic C. difficile carriage in humans, it is possible that changes in diet, host health, antibiotics, or other perturbations may lead to C. difficile outgrowth and expression of CDI. Thus, we advocate a careful, context-dependent interpretation of results when extrapolating the findings from animal models studies to clinical use.

## MATERIALS AND METHODS

### Materials.

Brain heart infusion (BHI) medium was purchased from BD Biosciences (Franklin Lakes, NJ). Reagents for DNA isolation were obtained from Qiagen (product no. 51504; Qiagen, Germantown, MD). Reagents for PCR were obtained from Quantabio (product no. 2200410; Quantabio, Beverly, MA). Standard diet (Laboratory Todent Diet 5001) and high carbohydrate diet (Low-Fat Control for Western Diet 9GT9) were purchased from TestDiet and irradiated prior to shipping. All mouse chow was stored at 4°C before use.

### C. difficile growth conditions and spore harvest.

C. difficile strain R20291 (RT027) was the kind gift of Dr. Nigel Minton (University of Nottingham, UK). C. difficile cells were allowed to sporulate by growing at 37°C for 7 days on BHI agar plates (Bacto) supplemented with 2% yeast extract, 0.1% l-cysteine HCl, and 0.05% sodium taurocholate. Plates were incubated in a Coy vinyl anaerobic chamber (Coy Lab Products, MI) containing 10% CO_2_, 10% H_2_, and 80% N_2_. Bacterial cells and spores were harvested from plates by washing with ice-cold, autoclaved, and deionized water, and gentle scraping. Vegetative cells and spores were pelleted by centrifugation and resuspended in fresh deionized water for a total of three wash cycles. Pellets were then centrifuged through a 20%-to-50% HistoDenz gradient (Sigma-Aldrich, St. Louis, MO) at 18,200 × *g* for 30 min with no brake to purify spores from cell debris. Pelleted spores were transferred to a clean centrifuge tube and washed with autoclaved deionized water five times. Schaeffer-Fulton-staining and phase-contrast microscopy were performed to ensure >95% purity of the harvested spores. The resulting purified spores were stored at 4°C until administration.

### Animals.

Female Syrian golden hamsters were purchased from Charles River (Wilmington, MA) and acclimated for a week in the animal facility prior to the experiments. Autoclaved bedding, water, and feed were used in all procedures. The Institutional Animal Care and Use Committee (IACUC) at the University of Nevada, Las Vegas, reviewed and approved this study (R0914-297). All experiments were performed according to the National Institutes of Health guidelines in the Guide for Care and Use of Laboratory Animals. Experimental animals were 5 to 8 weeks old.

### Treatment groups, induction, and monitoring of CDI, and sample collection.

A total of 30 hamsters were housed in individual cages and fed a standard lab diet for 6 days (until day −14) (Fig. 1a). Hamsters were then randomly assigned into three groups of 10 hamsters; two groups were fed a standard lab diet (standard lab diet and standard lab diet [-CDI]), and one group received a high-carbohydrate diet from day −14 onward for the remainder of the experiment (high-carbohydrate diet). Irradiated hamster chow was purchased from TestDiet and stored at 4°C before use. These are the exact same diets used previously for experiments in a mouse model of CDI (18). Diets and autoclaved water were given *ad libitum*. The hamsters in the high-carbohydrate diet and standard lab diet groups were given a single dose of clindamycin dissolved in autoclaved DI water (30 mg/kg) via oral gavage on day −1, challenged with 10^2^ CFU of C. difficile R20291 spores by oral gavage on day 0, and given vancomycin dissolved in autoclaved DI water (1 mg/mL) via oral gavage on days 0 to 2. Clindamycin is one of the antibiotics that is most commonly implicated in human gut dysbiosis and subsequent CDI and it is often used in animal models of CDI. Vancomycin was used to prevent non-CDI clindamycin-associated colitis (30), which led to death of animals before C. difficile challenge in pilot experiments, and to modulate CDI severity (23). The hamsters in the standard lab diet (-CDI) group did not receive any antibiotic treatment and were not infected with C. difficile spores. All hamsters were monitored daily for signs of CDI for 14 days postinfection, weighed twice daily after challenge inside a biosafety cabinet, and euthanized 14 days postinfection, or as soon as hamsters showed any CDI signs (diarrhea/increase in soiled bedding), so that no animals experienced unrelieved pain or distress, as required by IACUC-approved protocols. Fecal samples were collected beginning at day −20 and for the duration of the 34-day experiment as indicated in Fig. 1a, and archived for microbiome analysis at −80°C.

### DNA extraction and 16S rRNA gene amplicon sequencing.

DNA was extracted from fecal samples using the QIAamp Fast DNA Mini Stool Kit and quantified using a NanoDrop 1000 Spectrophotometer. Extracted DNA was sent to Argonne National Laboratory (Lemont, IL, USA) where the V4 region of the 16S rRNA gene was amplified from each DNA sample using the modified primers 515F: GTGYCAGCMGCCGCGGTAA and 806R: GGACTACNVGGGTWTCTAAT (56, 57). A unique forward primer containing 12 bp barcode was used to facilitate multiplexing of the samples (58). Next, cleaned PCR products were quantified and pooled at equimolar concentrations for paired-end sequencing (151 bp × 12 bp × 151 bp) on the Illumina MiSeq platform.

### 16S rRNA gene amplicon data processing.

Raw 16S rRNA gene amplicon sequences were imported into QIIME 2 (version 2018.6) (59) and demultiplexed using the sample-specific barcodes. Demultiplexed reads were denoised and dereplicated to obtain an amplicon sequence variants (ASVs) table using “dada2-denoise-paired” plugin. The ASV table was rarified at 8,384 sequences per sample and ASVs were classified to the lowest possible taxonomic rank using QIIME’s feature-classifier plugin and the Silva 132 99% OTUs full-length sequences (60) and was purged of ASVs assigned to mitochondria, chloroplasts, or were unidentified at the domain level. The resulting filtered ASV table with the taxonomic assignment, ASV phylogenetic tree, and associated metadata were imported in R (3.5.0) (https://www.R-project.org/) for further analyses using phyloseq (version 1.25.2) (61) and vegan (version 2.5.2). After quality filtering, there was a mean of 30,085 reads per sample, after rarefying the ASV table to 8,384 sequences per samples, a total of 410 samples were retained.

### Microbial diversity and statistical analyses.

To estimate alpha diversity, observed ASVs, Shannon, and Simpson diversity indices were calculated. To test if there were significant differences in alpha diversity in response to diet, antibiotic administration, and CDI, two-way mixed ANOVA with Bonferroni corrections were performed to conduct intragroup daily comparisons of alpha diversity indices with correction for multiple comparisons in SPSS (version 25). Similarly, two-way mixed ANOVAs followed by Tukey’s HSD tests were used to compare different groups on the same day. A few data points are missing data due to death of three hamsters in the standard lab diet cohort and eight hamsters in the high-carbohydrate diet cohort. The resulting analysis was visualized using ggplot2 (version 3.0.0) and modified in Inkscape. NMDS was performed based on Bray-Curtis dissimilarity to examine relationships between the samples with respect to diet, antibiotic treatment, and CDI over the course of experiment, and hypotheses were tested using ANOSIM. Further, similarity percent (SIMPER) analysis was performed to identify ASVs that contributed to 30% of the observed differences in microbial communities during the study (62).

One animal, hamster #9 in the standard lab diet group was shown to be a statistical outlier in nearly all analyses since it showed no apparent microbiome disruption (e.g., alpha diversity, Fig. S1) and was removed from figures and statistical calculations. Similar host-specific variability has been observed in diet-microbiome studies (63, 64) and may be related to rejection of the clindamycin gavage in our study.

### Data availability.

Files containing the original unfiltered sequences are available from the NCBI-SRA under accession number PRJNA766033.
