# Supplementary material for: A High-Carbohydrate Diet Prolongs Dysbiosis and Clostridioides difficile Carriage and Increases Delayed Mortality in a Hamster Model of Infection
Source: Microbiol Spectr. 2022 Jun 16;10(4):e01804-21. doi: 10.1128/spectrum.01804-21 (PMC9431659; doi:10.1128/spectrum.01804-21)
Supplement: Supplemental file 1 — Supplemental material. Download spectrum.01804-21-s0001.pdf, PDF file, 0.4 MB [file spectrum.01804-21-s0001.pdf]

Supplementary Material

Title: A high-carbohydrate diet prolongs dysbiosis and *Clostridioides difficile* carriage and increases delayed mortality in a hamster model of infection

Authors: Shrikant S. Bhute[1#], Chrisabelle C. Mefferd[1,2#], Jacqueline R. Phan[3], Muneeba Ahmed[1,4], Amelia E. Fox-King[1,4], Stephanie Alarcia[1], Jacob V. Villarama[1,5], Ernesto Abel-Santos*[3,6], and Brian P. Hedlund*[1,6]

Affiliation:

[1] School of Life Sciences, University of Nevada, Las Vegas, Las Vegas, NV, 89154

[2] School of Public Health, University of Nevada, Las Vegas, Las Vegas, NV, 89154

[3] Department of Chemistry and Biochemistry, University of Nevada, Las Vegas, Las Vegas, NV, 89154

[4] College of Osteopathic Medicine, Touro University, Nevada, Henderson, NV 89014

[5] Kirk Kerkorian School of Medicine, University of Nevada, Las Vegas, Las Vegas, NV, 89102

[6] Nevada Institute of Personalized Medicine, University of Nevada, Las Vegas, Las Vegas, NV, 89154

Corresponding authors:

* ernesto.abelsantos@unlv.edu, brian.hedlund@unlv.edu

# Authors contributed equally.

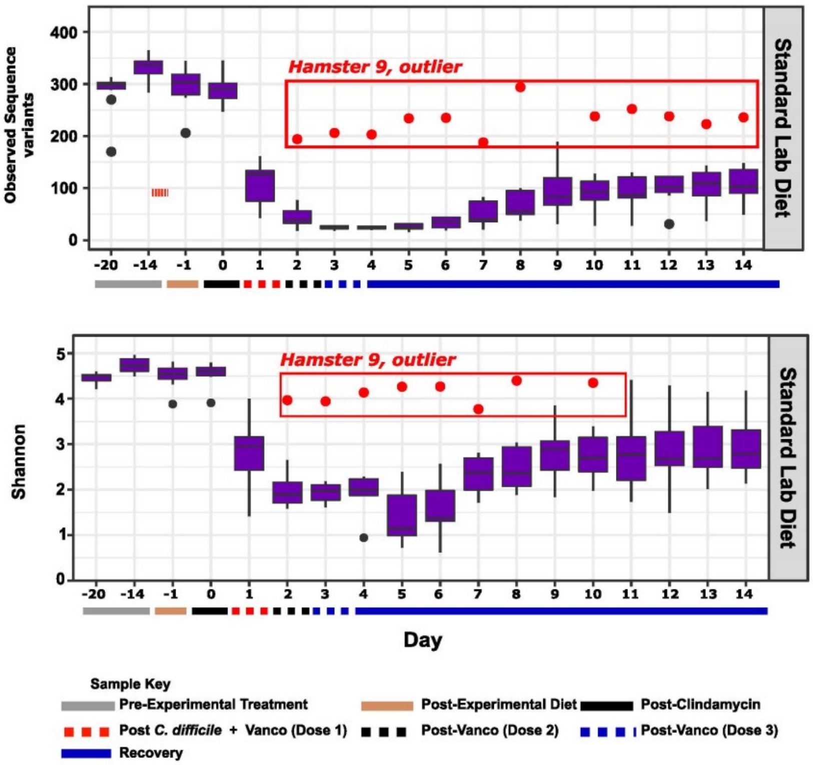

Figure S1. Evidence of a statistical outlier. Observed sequence variants and Shannon Diversity plots for the timeline of the experiments, showing that hamster #9 in the Standard Lab Diet Group did not experience dysbiosis, as evidenced by hamster #9 being a statistical outlier at every timepoint from Day 2 to Day 14 (observed sequence variants, top) or at every timepoint from Day 2 to Day 10 (Shannon Diversity, bottom). These data were used to justify removal of this animal from all other analyses.

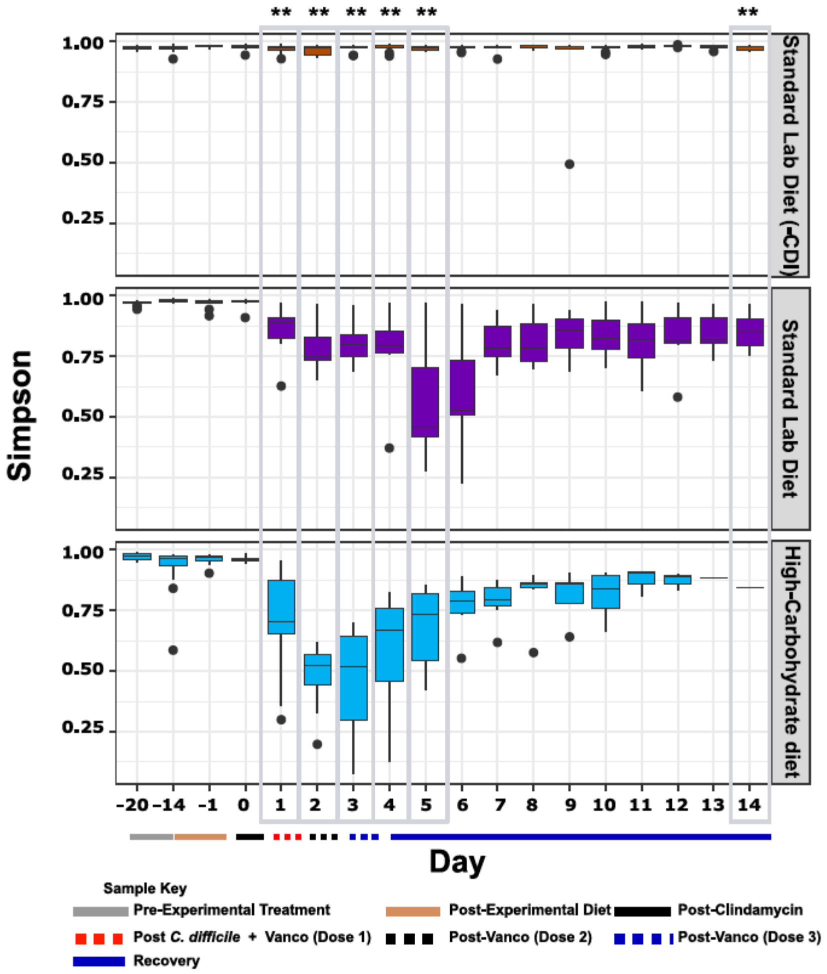

Figure S2. Effect of diet on Simpson's evenness throughout CDI course. Simpson's evenness values were calculated for uninfected hamsters fed the standard lab diet (brown, n = 10), infected hamsters fed the standard lab diet (purple, n = 9), and infected hamsters fed the high-carbohydrate diet (blue, n = 10). Administration of experimental diets (solid tan line, x-axis), timepoints after antibiotics (dashed lines, x-axis) and *C. difficile* challenge (black line, x-axis) are indicated. Gray boxes highlight comparisons between groups after a change in diet on Day 19, antibiotic treatments on Days 20-23, post-infection on Days 24 and 25, and recovery on Days 34. Black dots above and below boxplots represent outliers. (*) Indicates significant ($p < 0.05$) loss of diversity in within-group pairwise comparisons shown by the brackets. Gray boxes highlight comparisons between groups after a change in diet on Day -1, antibiotic treatments on Days 0-3, post-infection on Days 4 and 5, and recovery on Days 14. (**) Significant ($p < 0.05$) difference between groups on a given day (repeated measures mixed ANOVA followed by Tukey's HSD test).

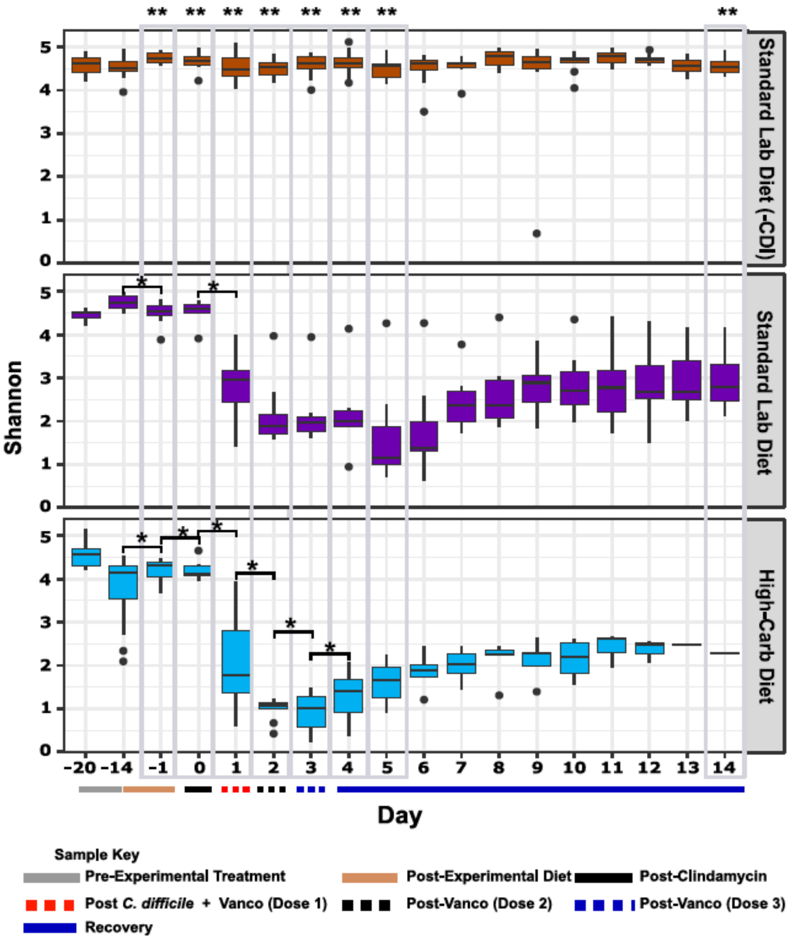

Figure S3. Effect of diet on Shannon diversity throughout CDI course. Shannon diversity values were calculated for uninfected hamsters fed the standard lab diet (brown, n = 10), infected hamsters fed the standard lab diet (purple, n = 9) and infected hamsters fed the high-carbohydrate diet (blue, n = 10). Administration of experimental diets (solid tan line, x-axis), timepoints after antibiotics (dashed lines, x-axis) and *C. difficile* challenge (black line, x-axis) are indicated. Gray boxes highlight comparisons between groups after a change in diet on Day 19, antibiotic treatments on Days 20-23, post-infection on Days 24 and 25, and recovery on Days 34. Black dots above and below boxplots represent outliers. (*) Indicates significant ($p < 0.05$) loss of diversity in within-group pairwise comparisons shown by the brackets. Gray boxes highlight comparisons between groups after a change in diet on Day -1, antibiotic treatments on Days 0-3, post-infection on Days 4 and 5, and recovery on Days 14. (**) Significant ($p < 0.05$) difference between groups on a given day (repeated measures mixed ANOVA followed by Tukey's HSD test).

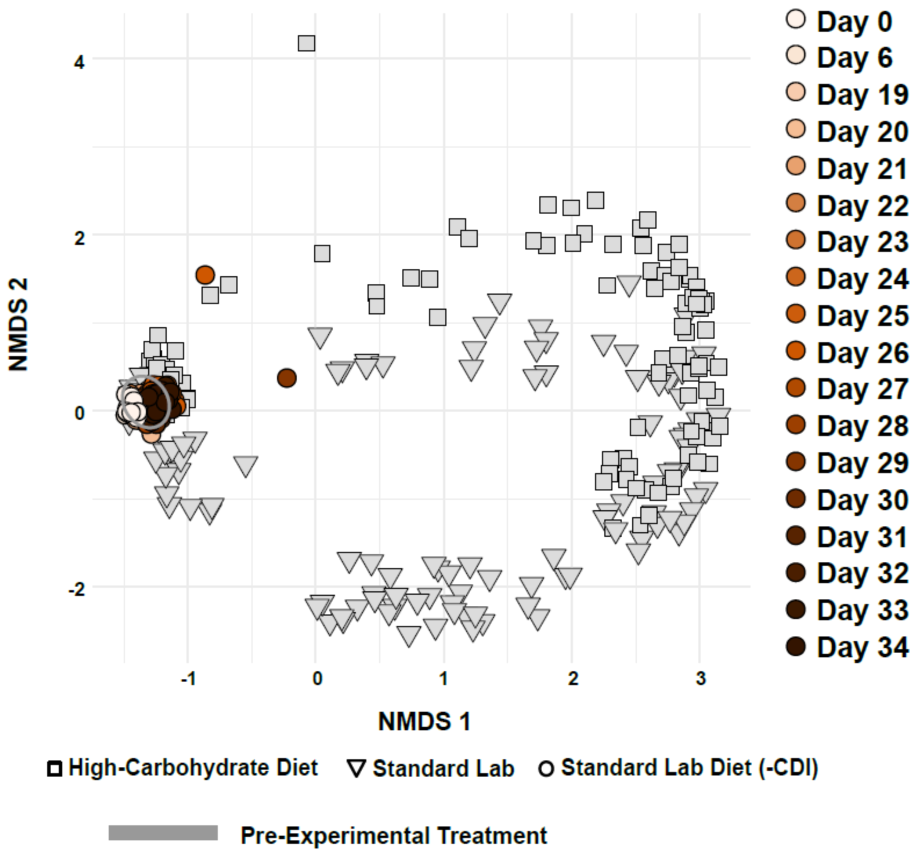

Figure S4. NMDS analysis based on Bray-Curtis dissimilarity (Standard Lab Diet -CDI). Displayed is a visualization of the same data shown in Figure 4 and highlights the analysis for uninfected hamsters fed the standard lab diet (tan, circles). Colors are shaded to show time progression through the experiment. The ellipse represents the standard errors of the mean (95% confidence) for samples associated with the standard lab diet without any treatments for the course of the experiment.
